# Identifying and addressing psychosocial determinants of adherence to physical distancing guidance during the COVID-19 pandemic – project protocol

**DOI:** 10.12688/hrbopenres.13090.2

**Published:** 2020-12-14

**Authors:** Hannah Durand, Simon L. Bacon, Molly Byrne, Eanna Kenny, Kim L. Lavoie, Brian E. McGuire, Jenny Mc Sharry, Oonagh Meade, Robert Mooney, Chris Noone, Laura L. O'Connor, Kate O'Flaherty, Gerard J. Molloy

**Affiliations:** 1 School of Psychology, National University of Ireland, Galway, Galway, H91 EV56, Ireland; 2 Montreal Behavioural Medicine Centre, Hôpital Sacré-Cœur de Montréal, Montreal, Quebec, QC H4J 1C5, Canada; 3 Department of Health, Kinesiology & Applied Physiology, Concordia University, Montreal, Quebec, QC H4B 2A7, Canada; 4 Department of Psychology, University of Quebec at Montreal, Montreal, Quebec, QC H2L 2C4, Canada; 5 Communications, Department of Health, Government of Ireland, Dublin, D02 XW14, Ireland; 6 Health and Wellbeing, Department of Health, Government of Ireland, Dublin, D02 XW14, Ireland

**Keywords:** COVID-19, SARS-CoV-2, social distancing, physical distancing, behaviour change

## Abstract

Optimising public health physical distancing measures has been a critical part of the global response to the spread of COVID-19. Evidence collected during the current pandemic shows that the transmission rate of the virus is significantly reduced following implementation of intensive physical distancing measures. Adherence to these recommendations has been poorer than adherence to other key transmission reduction behaviours such as handwashing. There are a complex range of reasons that are likely to predict why people do not or only partially adhere to physical distancing recommendations. In the current project we aim to address the following research questions: (1) What are the psychosocial determinants of physical distancing for the general public and for key socio-demographic sub-groups (e.g., young adults, older adults, etc.)?; (2) Do current Government of Ireland COVID-19 physical distancing communications address the determinants of physical distancing?; and (3) How can communications be optimised and tailored to sub-groups to ensure maximum adherence to guidelines? These will be addressed by conducting three work packages (WPs). In WP1, we will work closely with the iCARE international study, which includes a large online survey of public responses to measures established to reduce and slow the spread of COVID-19, including physical distancing. We will analyse Irish data, comparing it to data from other countries, to identify the key psychosocial determinants of physical distancing behaviour. This will be followed by a qualitative study to explore in depth the barriers and facilitators of physical distancing behaviour among the Irish public (WP2). In WP3, we will conduct a content analysis and evidence mapping of current government messaging around physical distancing, to ensure the findings from this research feed into the development of ongoing communication and future messaging about physical distancing.

## Introduction

Since its identification in December 2019, the spread of severe acute respiratory syndrome coronavirus 2 (SARS-CoV-2) has resulted in an ongoing global coronavirus disease 2019 (COVID-19) pandemic. In the absence of an effective vaccine, the key to halting the rapid spread of COVID-19 is public adherence to a range of public health behaviour-based prevention measures. One of the most powerful of these is *physical distancing* ( Anderson *et al.*, 2020; Islam *et al.*, 2020). In public health, physical distancing (previously referred to as *social distancing*; Harris *et al.*, 2020; Kumar, 2020) is a set of measures intended to prevent the spread of a contagious disease by maintaining a physical distance between people and reducing the number of times people come into close physical contact with one another ( Harris *et al.*, 2020). From an epidemiological perspective, the goal of these distancing measures is to decrease the effective reproduction number, or R, (i.e., the average number of people an infected person infects in turn) to below 1, whereby the outbreak will begin to shrink. Once this number is reduced to well below 1, governments can begin to ease more stringent restrictions (e.g., school and workplace closures) while keeping the number of new cases stable. 

Physical distancing behaviours (specifically: keeping a distance of at least two metres from others outside of your household; limiting one’s number of close contacts; avoiding social gatherings) appear to be more difficult for many members of the public to initiate and maintain than other key transmission reduction behaviours such as handwashing. For example, a poll of 1,460 adults in Ireland conducted in March 2020, following the introduction of physical distancing measures by the Irish government, revealed that only 54% of participants reported sitting further apart from others more than usual, whereas 90% indicated that they were washing their hands more ( Amárach Research, 2020). Similar behavioural patterns have been observed in the UK ( Atchison *et al.*, 2020) and the USA ( de Bruin & Bennett, 2020). In the context of the unprecedented health, social and economic crisis that COVID-19 presents, one in which the global need for adherence to public health policies is paramount, our understanding of the determinants of adherence to physical distancing guidelines is critical for effective policy planning and communication.

There are multiple possible psychosocial variables that are likely to account, in part, for the resistance to physical distancing-related behaviour change ( Atchison *et al.*, 2020; Williams *et al.*, 2020; Wirz *et al.*, 2020). These diverse factors can be usefully summarised by two complementary theoretical approaches: (1) the COM-B (Capability, Opportunity, Motivation-Behaviour) model ( Michie *et al.*, 2011); and (2) the Health Belief Model ( Rosenstock *et al.*, 1988), which, together, provide a framework for understanding the psychological, behavioural, social, and environmental factors that predict human behaviour change and adaptation. Foremost among the factors that are likely to include the public's beliefs about COVID-19 and about physical distancing as a countermeasure to reduce disease transmission. These illness and treatment beliefs ( Hagger *et al.*, 2017) about COVID-19 and physical distancing are likely to vary according to key demographics and membership of certain “at-risk” groups, for example, older age groups ( Atchison *et al.*, 2020) and people who are immunocompromised. In addition to these reflective cognitive factors, there are automatic psychological processes that are likely to make physical distancing behaviour difficult to adopt. For example, automatic responses to shake hands or to move into the close physical proximity of friends, neighbours and colleagues when we see them are difficult to inhibit, given that they are largely non-conscious behaviours that have become highly habitualised ( Hollands *et al.*, 2016; Marteau *et al.*, 2012).

In the current work we aim to identify key modifiable determinants of adherence to physical distancing and to examine whether current government COVID-19 communications optimally target these determinants. Results from this programme of work will be fed directly to the National Public Health Emergency Team (NPHET) COVID-19 Communications and Behavioural Advisory Group (formerly the Subgroup on Behavioural Change) to ensure that our findings inform and impact on the development and refinement of physical distancing-related public health communications in the Republic of Ireland.

## Protocol

**Figure 1.**
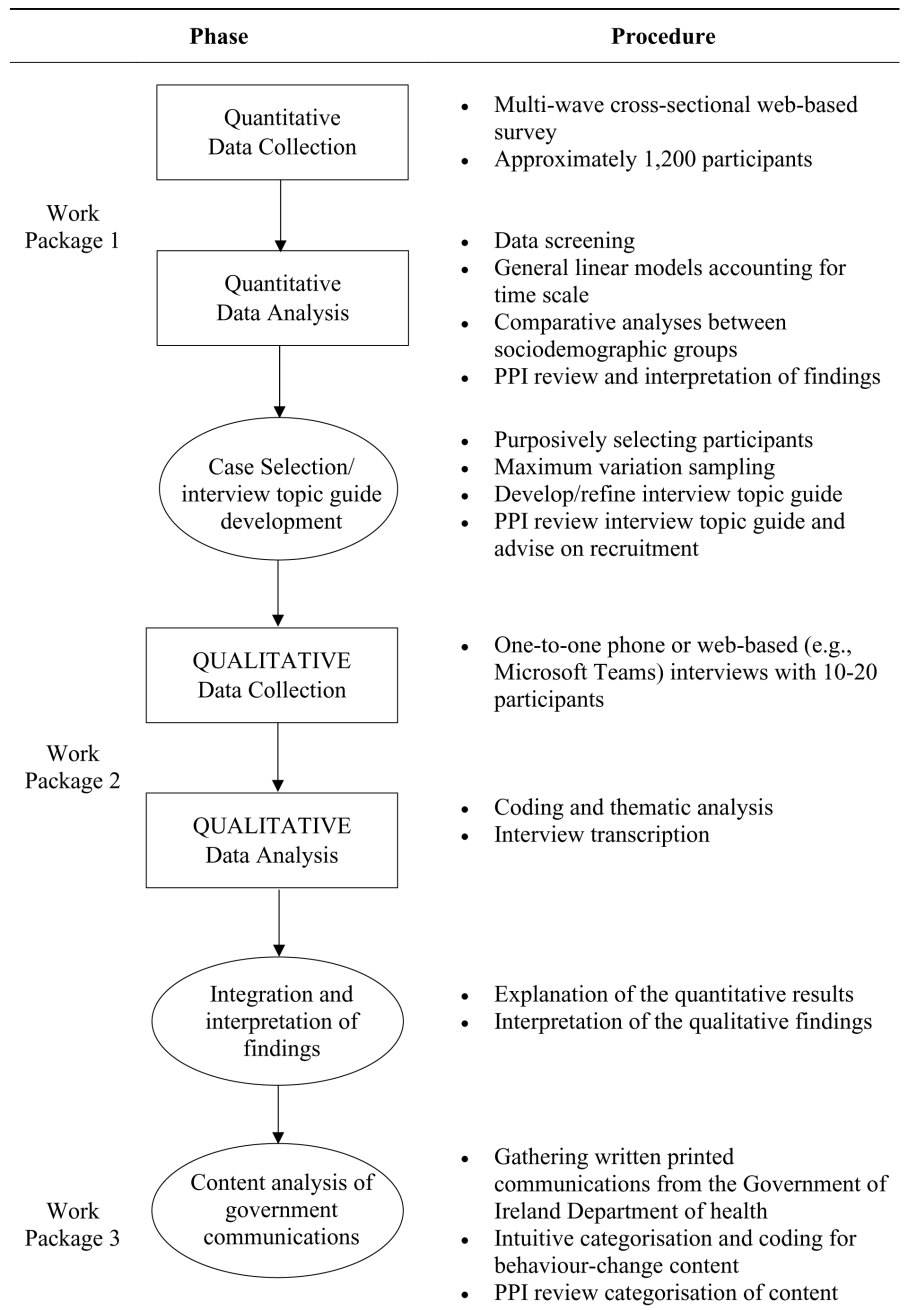
Visual model of the three work packages.

The current project consists of three related work packages (WPs) that aim to identify and address psychosocial determinants of physical distancing behaviour. The protocol for each WP is described in detail below. A visual model of the three WPs is presented in Figure 1. The project is registered with the Open Science Framework ( Durand *et al.*, 2020).

### WP1: Quantitative investigation into determinants of adherence to physical distancing guidelines

***Study overview.*** WP1 constitutes analysis of quantitative observational data collected as part of the International Assessment of COVID-19-related Attitudes, Concerns, Responses and Impacts in Relation to Public Health Policies (iCARE) study. The iCARE study, led by Kim Lavoie and Simon Bacon of the Montreal Behavioural Medicine Centre (MBMC), is an ongoing international longitudinal study which includes an online survey of public responses to measures established to reduce and slow the spread of COVID-19, including physical distancing. The online survey, which commenced in March 2020, will be circulated in waves each approximately five weeks apart and across multiple countries, including Ireland. This data will be used to determine which strategies, launched where, when, and for whom were most (and least) associated with adherence and most (and least) effective at reducing infection rates and mortality and minimising impacts, in order to provide data-driven recommendations to governments on how to optimise policy and communication strategies to improve policy adherence and health, economic, and quality of life outcomes. Full details of the iCARE study are described in Bacon & Lavoie (2020).

***Study aim.*** To examine the sociodemographic (e.g., age, sex, gender, ethnicity, parental status, employment/student status, built environment, healthcare system factors), psychological (e.g., COVID-19 attitudes, beliefs and concerns), behavioural, physical/mental health, and economic determinants of adherence to physical distancing guidance in Ireland.

***Study design.*** The current study forms part of the iCARE study led by the MBMC. iCARE is an international multi-wave cross-sectional observational cohort study. 

***Sample selection and recruitment.*** iCARE survey data is being collected in waves every five weeks from March 2020 to at least January 2021 using: (i) convenience snowball sampling; and (ii) parallel representative sampling. First, the online survey, created using the LimeSurvey ^©^ online survey tool, will be distributed through various international channels to reach as many people around the world as possible. These channels include professional networks, associations, and societies; schools and universities; hospitals and health networks; community organisations; social media; and personal contacts. Participation in the iCARE study will be voluntary and no personal identifying information will be collected. The survey will be distributed in each participating country until local prevention measures are lifted and/or the WHO retracts the declaration of global health emergency. There will be no compensation for people in the convenience sample who complete the survey. In tandem, Amárach Research panel provision services will be utilised to target a large, nationally representative sample of Irish respondents. This combination of approaches will ensure maximum variation in the sample in terms of key demographic and socioeconomic factors. Given that the iCARE study is already underway, with data collection for Waves 1 and 2 already completed, targeted recruitment efforts will be focused on Waves 3 (June – July 2020) and 4 (July – August 2020). The primary disadvantage of this approach is that it does not allow for deviations from the international study protocol in response to the specific trajectory of the pandemic in Ireland. However, connecting with the existing iCARE survey study, as opposed to creating and circulating a new survey locally, will help to reduce participant burden and fatigue among the general public and to minimise research waste ( Glasziou *et al.*, 2020), and allow us to contextualise Irish responses within the broader international iCARE sample. 

***Data collection.*** Responses to the iCARE survey will be collected online. Although online surveys may limit participation from individuals without access to the internet ( Szolnoki & Hoffmann, 2013), the advantages of this approach have been shown to outweigh the disadvantages in terms of external validity ( Heen *et al.*, 2014). Furthermore, as of 2018, it is estimated that 89% of households in Ireland have access to the Internet at home ( Central Statistics Office, 2018), meaning that the resultant risk of bias from using this approach is relatively low. Individuals without access to the Internet will be afforded the opportunity to participate in WP2 (described below), reducing the overall risk of bias in findings from the current programme of research.

**Figure 2.**
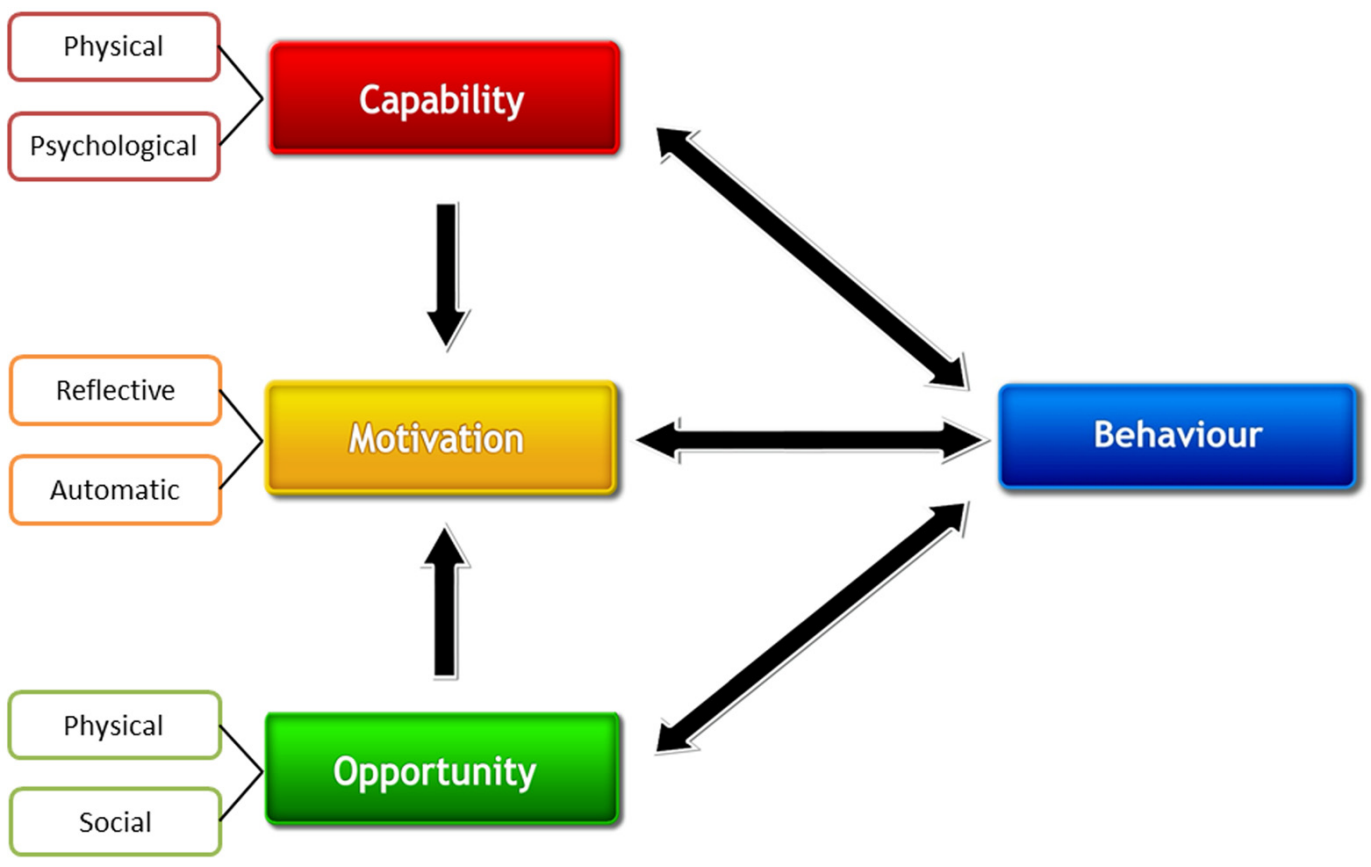
The COM-B model ( Michie *et al*., 2011).

A questionnaire tool was designed specifically for this study. Due to the unavailability of validated scales, questionnaire items were determined in line with current global COVID-19 prevention policies and health psychology theory, specifically the COM-B (Capability, Opportunity, Motivation-Behaviour) model ( Michie *et al.*, 2011), a framework for understanding behaviour as an interaction between capability, opportunity, and motivation factors, and the Health Belief Model ( Rosenstock *et al.*, 1988), a social cognitive health behaviour change model developed to explain and predict health-related behaviours. The COM-B model ( Figure 2) posits that behaviour is the result of the interaction between capability, opportunity, and motivation components. Each of these components is further broken down: capability can be psychological (knowledge) or physical (skills); opportunity can be social (societal influences) or physical (environmental resources); and motivation can be automatic (emotion) or reflective (beliefs, intentions). It provides a useful framework for the identification of potential intervention functions and policy categories, which makes it a particularly useful framework to underpin research with public health implications. The Health Belief Model posits that an individual’s belief in the personal threat of an illness or disease together with their belief in the effectiveness of the recommended health behaviour will predict the likelihood of that individual adopting that behaviour. It derives from psychological and behavioural theory with the foundation that the two components of health-related behaviour are the desire to avoid illness and the belief that a specific health action will prevent illness. The six core constructs of the Health Belief Model are perceived susceptibility, severity, benefits, and barriers, and cues to action and self-efficacy. The Health Belief model is one of the most widely used theories in health behaviour research ( Glanz & Bishop, 2010).

The online survey includes 31 questions (92 items) assessing socio-demographics, occupational status, health status, general health behaviours, awareness of local health authority prevention measures, attitudes and perceived concerns about the virus, and adherence to prevention measures instituted in the respondent’s country/region. Of these 92 items, 13 relate to physical distancing behaviours (e.g., Staying at least 2 metres away from others; Working from home; Self-isolating; Avoiding social gatherings). The full questionnaire tool is publicly available via the Open Science Framework ( Lavoie & Bacon, 2020). The survey takes approximately 15–20 minutes to complete and all responses are anonymous.

***Sample size.*** A panel of approximately 1000 adult participants, representative of the national population in the Republic of Ireland in terms of age, sex, geography and socioeconomic status, will be recruited to the study through Amárach panel provision services.

***Data analysis.*** The proposed analyses will focus on the nationally representative sample of Irish respondents collected in Wave 3 of the iCARE study. This is an exploratory study; therefore, hypotheses are not specified *a priori*. The aim of this analysis will be to estimate the relative strength of a range of theoretically informed predictors on adherence to physical distancing guidelines. Data from Ireland will be analysed locally by the HRB-funded research team to evaluate determinants of adherence to Irish guidelines, and to facilitate cross-country comparison. The primary analyses will be general linear models using multilevel modelling software. Of particular interest are comparative analyses between sociodemographic groups. Supplementary analyses may be conducted in response to ongoing changes in public health information and restrictions that pertain at the time of analyses in Ireland.

### WP2: Qualitative exploration of barriers and facilitators of adherence to physical distancing guidelines

***Study overview.*** A qualitative study will be carried out to explore in greater depth the barriers and facilitators of adherence identified in sub-groups (i.e., various sociodemographic and at-risk groups), from the quantitative analyses in WP1, and to provide more in-depth insight into the role of Irish context-specific determinants of behaviour beyond the scope of the quantitative iCARE study. WP2 will constitute one of the first qualitative studies of adherence to physical distancing measures during the COVID-19 pandemic, which will serve to provide vital insight into people’s feelings, opinions, and experiences of physical distancing. Participants will be invited to participate in a semi-structured one-to-one phone/web-based interview via targeted social media and email advertisements. Interviews will be audio recorded, transcribed verbatim and analysed using thematic analysis. 

***Study aim.*** To qualitatively explore barriers and facilitators of physical distancing in the context of the COVID-19 pandemic. 

***Study design.*** A qualitative interpretive design will be employed.

***Sample selection and recruitment.*** A purposive sampling strategy involving the deliberate choice of participants due to specific qualities that they possess ( Etikan *et al.*, 2016) will be employed in an effort to achieve maximum variation in perspectives on physical distancing. We will aim to recruit participants that differ in key sociodemographic factors (e.g., age, gender, socioeconomic status) and COVID-19 experience (e.g., diagnosed and/or hospitalised with COVID-19, no personal experience of COVID-19 symptoms). Relevant sociodemographic and COVID-19 factors will be selected based on variables associated with greater or poorer adherence to physical distancing guidance as identified in WP1, as well as findings from other relevant published work including preprints (e.g., sources identified in an ongoing scoping review by Noone *et al.* (2020)). Participants will be recruited via targeted social media and email advertisements circulated via community groups, professional organisations and personal networks, as required. Initial analysis will guide decision making regarding subsequent sampling (e.g., to increase representation of various sub-groups of interest); that is, if initial recruitment efforts result in an over- or under-representation of certain demographic groups then participants from those under-represented groups will be specifically targeted to ensure maximum variation in the sample.

***Sample size.*** Although there are no widely accepted formulae for calculating required sample sizes for qualitative studies, it has been recommended that 10–20 interview participants are recruited for studies of this kind ( Braun & Clarke, 2013). Within the scope of these guidelines, the final sample size will be informed by evaluation of data adequacy in terms of both the amount and variety required to answer the research question ( Guest *et al.*, 2020; Vasileiou *et al.*, 2018), and representation of key sociodemographic groups.

***Data collection.*** Semi-structured interviews will be conducted with individual participants via phone or a secure web-based platform (e.g., Microsoft Teams), depending on participant needs and preferences, by members of the research team. Semi-structured interviews were chosen for this work given the appealing balance between structure and flexibility they allow. A topic guide of open-ended questions will be used to flexibly guide the interviews (see *Extended data;* Durand *et al.*, 2020). Electronic consent will be sought from all participants to prevent face-to-face contact and to maintain physical distancing. 

***Data analysis.*** Interview transcripts will be analysed using thematic analysis guidelines described by Braun & Clarke (2006). Thematic analysis was selected as the primary analytic approach as it is highly flexible, and provides a rich and detailed, yet suitably complex account of qualitative data ( Braun & Clarke, 2006; King, 2004). The analysis will be conducted from a subtle realist perspective ( Hammersley, 1992), which acknowledges the subjective nature of knowledge while maintaining a belief in the existence of an underlying reality that we attempt to represent through research ( Mays & Pope, 2000). A reflexive approach that acknowledges and considers the centrality of researcher subjectivity will be undertaken throughout the study ( Braun *et al.*, 2018). This will allow the researchers to consider and analyse how subjective and intersubjective elements influence the research process. This is particularly pertinent in the context of COVID-19 research, given the likely impacts the pandemic has had and will continue to have on the lives of the researchers undertaking this study. Interviews will be transcribed by members of the research team to facilitate the familiarisation and interpretive processes ( Bailey, 2008). NVivo 12 software ( QSR International, 1999) will be used to manage the data and to facilitate the thematic analysis. 

Considerations will be made to ensure WP2 is carried out rigorously and that the data and analysis are of sufficient quality, in accordance with Yardley (2000). The research team will adopt a systematic approach to participant recruitment and data collection to ensure the rigour and credibility of the study ( Lincoln & Guba, 1985; Polit & Beck, 2011). Each researcher involved in interviewing participants and analysing the data has qualifications and practical skills and experience in conducting qualitative research ( Patton, 1990). The reflexive process will be documented to provide a transparent record of decisions made throughout the study. The interview topic guide will be generated using existing empirical evidence and consultation with members of a Public and Patient Involvement (PPI) panel, described below. We will establish authenticity and confirmability using participant quotes and careful synthesis of perspectives to support the findings and to ensure any conclusions drawn are well grounded in the data. 

### WP3: Content analysis and evidence mapping of current government physical distancing communications

***Study overview.*** Current communications issued by the Government of Ireland Department of Health (DoH) and/or Health Service Executive (HSE), intended to change the public’s behaviour through persuasive communication ( Miller, 1980), will be independently collated and reviewed by two researchers using a theory- and evidence-based content analysis approach ( Carey *et al.*, 2019). Current messages will then be mapped onto the profile of adherence determinants identified in WP1 and WP2 to identify gaps in current physical distancing communications. Identifying gaps in current communications will allow for the development of new supplementary and sub-group tailored messages addressing evidence-based determinants of adherence behaviour.

***Study aim.*** To analyse the content of government physical distancing communications in light of the most recently available data on physical distancing to provide evidence-based recommendations to optimise future government messaging campaigns.

***Data gathering.*** Data in this instance refers to written printed communications from the Government of Ireland DoH in poster format. These may include posters intended for distribution via social media, news outlets, print media, signs on public streets, et cetera. Communications will be systematically gathered by the research team from the DoH official website. Any additional posters not available through the DoH website will be obtained from collaborators at the DoH and NPHET. Depending on the number of messages obtained, and the amount of duplication among them, a smaller subset of messages may be selected out for the content analysis.

***Categorisation and coding.*** Initially a novel intuitive categorisation process will be carried out on the sample material according to length/number of words, date of issue (with reference to the phase of easing of restriction measures; Phase 2, 3, etc.), target audience (general public versus specific sectors), et cetera. We will draw in part on previous work by Seppälä *et al.* (2018), which involved a content analysis of policy papers in Finland, to guide the categorisation. Messages will then be coded for behaviour-change content. Specifically, messages will also be coded for established behaviour change techniques using the Behaviour Change Technique Taxonomy Version 1 ( Michie *et al.*, 2013). These techniques will then be mapped onto one of the 26 Mechanisms of Action ( Carey *et al.*, 2019) using the Theories and Techniques tool. Disagreements between reviewer pairs will be resolved by consensus, or by consulting a third reviewer, on a case-by-case basis. Note that the final coding approach will be informed by consultation with our PPI panel (described below).

***Optimisation of future communications.*** A report of findings from WP3 identifying gaps in current communications and making recommendations to support the development of tailored messages addressing evidence-based determinants of adherence behaviour using established behaviour change techniques will be provided to the DoH and NPHET via the COVID-19 Communications and Behavioural Advisory Group.

### PPI

PPI in COVID-19-related research is crucial given the significant and far-reaching impact that the pandemic has had on all members of society. Consistent and meaningful research input from the public will be required to address the current crisis ( Murphy *et al.*, 2020). With this in mind, PPI will be implemented across all WPs in this project in collaboration with a PPI panel (described below). Given that the iCARE study is already underway, our panel’s involvement in WP1 will begin at the point of analysis, whereby members of the public will be asked to review and interpret the quantitative findings (note: researchers at the MBMC consulted with 150 collaborators from over 35 countries including researchers, clinicians, students, and members of the general public in the development and design of the iCARE study). In WP2, PPI partners will be invited to review the interview topic guide for its clarity and relevance, to advise on strategies to recruit a diverse and representative sample, with particular emphasis on recruiting participants from relevant sociodemographic groups (as identified through WP1 and the emerging scientific literature), and to contribute to the qualitative analysis process. In WP3, PPI partners will be invited to contribute to and validate the content analysis of current government messages and mapping of message content to barriers and facilitators identified in WPs 1 and 2. The exact nature of these PPI activities will evolve through discussion among the PPI panel and the research team.

We are currently recruiting a PPI panel to work with the project team to enhance its relevance, quality, and impact. We invited current and former PPI panellists from healthcare-related projects at NUI Galway. Additionally, the PPI Ignite @NUI Galway office (HRB-funded initiative to provide support and training in PPI to researchers and members of the public; see https://www.nuigalway.ie/ppi/) circulated this opportunity to interested members of the public. Finally, we asked community and university-based organisations to share information about this opportunity with their members.

We aim to recruit a diverse panel of eight PPI contributors from varied sociodemographic backgrounds. Panel members will meet six times over the duration of the project. Initial meetings will take place online due to COVID-19 physical distancing restrictions. PPI contributors will also have the option of participating in meetings by phone. It is likely that our recruited panel members will have varied levels of PPI experience. Preparatory training needed for PPI tasks will be provided by the study team. 

Details of PPI included in published research articles will be reported in line with the Guidance for Reporting Involvement of Patients and the Public Version 2 (GRIPP2) checklist ( Staniszewska *et al.*, 2017).

### Data management

All identifying data (i.e., audio recordings of interviews) will be stored on a password-protected computer prior to anonymisation. Once audio recordings have been transcribed and anonymised, original recordings will be destroyed. All regulations set by the Research Ethics Committee at NUI Galway will be observed, as well as General Data Protection Regulation (GDPR). 

### Ethical considerations

This research, particularly WP2, has the potential to cause distress given the far-reaching personal, economic, and social impacts of the COVID-19 pandemic, particularly for those who have direct experience of hospitalisation, bereavement, unemployment, or loneliness related to COVID-19. Participants will be aware in advance that they will be asked to discuss their experience of the pandemic and the related impacts on their life; therefore, they will be prepared for the potentially upsetting nature of the interview. All participants will be guided towards relevant supports in the community (e.g., general practice and community-based psychological support) by the research team. All researchers will have an experienced line manager/supervisor who will be available for ongoing advice and support in relation to carrying out the study. As surveys and interviews will be carried out remotely, there will be no risk of COVID-19 transmission as a consequence of taking part in this research.

Ethical approval has been granted for this work by the Research Ethics Committee at NUI Galway (Ref no.: HRB20-Apr-18). The iCARE study has been approved by the Human Research Ethics Committee of the Centre intégré universitaire de santé et de services sociaux (CIUSSS) du Nord-de-l’Île-de-Montréal (Ref no.: #2020-2099 / 03-25-2020).

### Study status

- WP1: Recruitment is currently underway.
- WP2: Refinement of the interview topic guide is underway.
- WP3: Gathering of current physical distancing communications is currently underway.
- PPI: Recruitment of PPI contributors is underway.

### Dissemination and knowledge exchange plan

Findings from this research will be of international relevance given the global impact of COVID-19. Ensuring rapid dissemination of results through a variety of channels is a priority to ensure that policy makers, researchers, and the public have access to the most up-to-date data available. This project will be carried out in line with principles of Open Science to ensure that the information gathered is freely accessible.

***Key national stakeholders.*** Dissemination and knowledge exchange will be promoted from the outset through involvement of key national policy partners from the DoH (RM and KOF) and PPI panel members as advisors in conduct of the research. Research outputs will be disseminated, translated, and shared primarily via the NPHET COVID-19 Communications and Behavioural Advisory Group, of which co-authors MB, RM, and KOF are members. The Communications and Behavioural Advisory Group publicly disseminates its findings via its website, which is regularly updated with reports and information. Any deliverables from the research will be promoted via social media channels, such as Twitter and Facebook.

***Academic dissemination.*** Research findings are of relevance to the international scientific and community as countries worldwide respond to the COVID-19 pandemic. The studies will be written up for publication in open-access, peer-reviewed journals, prioritising outlets which will facilitate a speedy publication timeframe. Preprint versions of the manuscripts will be posted on public preprint servers ( Kirkham *et al.*, 2020) before formal publication in scholarly journals. Relevant reporting guidelines will be utilised to ensure the completeness and transparency of the articles; specifically, findings from WP1 will be reported in line with the STrengthening the Reporting of OBservational studies in Epidemiology (STROBE) Statement ( von Elm *et al.*, 2008), and findings from WP2 will be reported in line with the COnsolidated criteria for REporting Qualitative research (COREQ) checklist ( Tong *et al.*, 2007).

***Dissemination to the public.*** All findings will be communicated to the public via lay summaries and graphics circulated through social media, email, University webpages, et cetera, in an effort to reach as wide a public audience as possible. Our PPI panel will be asked to contribute to the preparation of these communications to ensure that they are clear and accessible.

***Approach to data sharing.*** In line with HRB Policy on Open Access, we will ensure that all outputs and anonymised study data and/or analysis protocols are made openly accessible to the public via public repositories and/or by reasonable request (e.g., in the case of qualitative interview data that may be considered identifiable despite attempts at anonymisation). Any data to be shared publicly, such as data used in the generation of publications arising from the study, will be anonymised in advance and in accordance with NUI Galway Standard Operating Procedures for Research Quality. GDPR will be adhered to. The lead researchers on the international iCARE study are currently working on a process to make the global and country-level data open access. A data sharing agreement between the MBMC and the research team at NUI Galway is in place.

## Conclusion

Physical distancing is one of the best strategies that we have to slow the spread of COVID-19. However, keeping our distance from others is perhaps the most difficult and even unnatural behaviour that we must adopt. As restrictions on public services, commerce, and travel ease, in line with the Government of Ireland’s plan for living with COVID-19, maintaining physical distance will continue to play a vital role in ensuring that a rebound in transmission rates does not occur ( Kissler *et al.*, 2020). Understanding the complex factors involved in determining whether someone will or will not adhere to physical distancing guidelines, and addressing those factors in evidence-based, fully transparent, and fact-based public health campaigns ( Ghio *et al.*, 2020; Lewnard & Lo, 2020), is therefore of critical importance. Utilising both quantitative and qualitative data, which are distinct but complementary and commensurate, will serve to enhance our understanding of physical distancing behaviours. The large scale iCARE survey will allow us to gauge the attitudes and behaviours of a large nationally representative sample, while the qualitative study will allow us to drill down into key topics of interest in a much more in-depth way than a survey study can allow. Therefore this programme of work will provide high-quality accelerated evidence on the barriers and facilitators of physical distancing to support COVID-19 policy strategy and communication in the Republic of Ireland.

## Data availability

### Underlying data

No underlying data are associated with this article.

### Extended data

Open Science Framework: Identifying and addressing psychosocial determinants of adherence to physical distancing guidance during the COVID-19 pandemic. https://doi.org/10.17605/OSF.IO/TXS37 ( Durand *et al.*, 2020).

This article contains the following extended data:

- Physical Distancing Interview Topic Guide v1.pdf

Data are available under the terms of the Creative Commons Attribution 4.0 International license (CC-BY 4.0).
