# Supplementary material for: Identifying and addressing psychosocial determinants of adherence to physical distancing guidance during the COVID-19 pandemic – project protocol
Source: HRB Open Res. 2020 Dec 14;3:58. Originally published 2020 Aug 26. [Version 2] doi: 10.12688/hrbopenres.13090.2 (PMC7791346; doi:10.12688/hrbopenres.13090.2)
Supplement: Peer review history

# Peer review: Reviewer response for version 2

**DOI:** 10.21956/hrbopenres.14334.r28547

**Published:** 2021-01-07

**Authors:** Maik Bieleke, Leon Barton

**Affiliations:** 1 Department for Psychology of Development and Education, Faculty of Psychology, University of Vienna, Vienna, Austria; 2 Department of Psychology, University of Konstanz, Konstanz, Germany

The authors thoroughly addressed and satisfactorily responded to all our comments. We find the study protocol convincing and congratulate the authors for their great and important work.

Is the study design appropriate for the research question?

Yes

Is the rationale for, and objectives of, the study clearly described?

Yes

Are sufficient details of the methods provided to allow replication by others?

Partly

Are the datasets clearly presented in a useable and accessible format?

Not applicable

Reviewer Expertise:

Psychology, self-regulation, motivation, goals, behavior change.

We confirm that we have read this submission and believe that we have an appropriate level of expertise to confirm that it is of an acceptable scientific standard.

# Peer review: Reviewer response for version 2

**DOI:** 10.21956/hrbopenres.14334.r28546

**Published:** 2020-12-15

**Authors:** Catherine Darker

**Affiliations:** 1 Department of Public Health & Primary Care, Trinity College Dublin, Dublin, Ireland

Thank you to the research team for addressing the comments that I have made. I have no further comments to make. I wish you the best of luck with this important piece of research.

Is the study design appropriate for the research question?

Yes

Is the rationale for, and objectives of, the study clearly described?

Yes

Are sufficient details of the methods provided to allow replication by others?

Partly

Are the datasets clearly presented in a useable and accessible format?

Not applicable

Reviewer Expertise:

Health psychology, public health/population health, health behaviour change interventions, health promotion

I confirm that I have read this submission and believe that I have an appropriate level of expertise to confirm that it is of an acceptable scientific standard.

# Peer review: Reviewer response for version 1

**DOI:** 10.21956/hrbopenres.14191.r27886

**Published:** 2020-09-28

**Authors:** Maik Bieleke, Leon Barton

**Affiliations:** 1 Department for Psychology of Development and Education, Faculty of Psychology, University of Vienna, Vienna, Austria; 2 Department of Psychology, University of Konstanz, Konstanz, Germany

Durand *et al*. propose a quantitative and qualitative approach to understand the psychological and sociodemographic determinants of adherence to physical distance, as well as the degree to which government actions to increase physical distancing address these determinants adequately. As such, this research will likely contribute in a significant way to the scientific understanding and practical implementation of physical distancing measures during the response to COVID-19. While we endorse the overall approach of the authors, we identified some issues the authors should address to further strengthen their approach and its theoretical foundation.

1. “This is an exploratory study; therefore, hypotheses are not specified a priori.”
  Why are no hypotheses specified? The authors outline empirical evidence regarding the variables used in their study and rely on two theoretical models to develop the research design, both of which could serve as a basis for formulating hypotheses. Moreover, the authors already seem to have hypotheses in mind when they refer to COVID-19-related beliefs/concerns and “at-risk groups”. 
2. The authors acknowledge the importance of automatic psychological processes as determinant for physical distancing adherence (e.g., habits). Yet, they solely refer to models addressing more reflective processes (e.g. beliefs) and remain silent about how these automatic processes are taken into account. This should be addressed, or explicated as a limitation of the proposed research. 
3. The authors describe very briefly two well-established models which contributed to their work in significant way. However, it might be useful if the authors outline the two models in a more detailed and a more distinctive manner (e.g. how the perceived severity of COVID-19 impacts physical distancing adherence). Especially the COM-B model should be treated in more detail because it is merely mentioned and not further explicated or discussed. As this research might be important for different fields of research (e.g. medicine, politics, sociology), a broader introduction of psychological theorizing seems appropriate. 
4. Please operationalize the term “physical distancing” in a more distinct and detailed manner. Different measures can be implemented in order to keep physical distance among citizens. How is this variable measured? For instance, is it a single question or a questionnaire? And if it is a questionnaire, how will an overall physical distancing score be determined? 
5. The rationale for WP 2 remains somewhat fuzzy. The authors state that it is carried out to “explore in greater depth the barriers and facilitators of adherence” in an effort to complement the quantitative analyses in WP 1. However, it is unclear what the specific insights are that the authors hope to gain from the respondents in WP 2. What would be an outcome that goes beyond the quantitative measures? Alternatively, what do the quantitative measures miss that can only be addressed in the proposed interviews? This should be made more explicit, especially because WP 2 currently seems to make the smallest contribution to attaining the overall study goals. 
6. The sample size considerations for WP 2 are currently not convincing. Answers from only 10-20 respondents could be biased by idiosyncratic experiences that might be hard to generalize to the public. On the other hand, it seems that the authors plan to recruit more participants if needed. They should specify the rules for extending/stopping data collection.
7. “This selection procedure, if necessary, will be informed by previous work of a similar nature and discussion among the research team to ensure that any selection biases in selecting public health for content analyses are limited.”
  Please outline the selection procedure in more detail or provide a reference where the procedure is described.
8. In WP 3, the authors should be more specific about how they intend to map government actions and the psychosocial determinants of adherence studied in WP 1 & 2. For instance, will this be a quantitative or a qualitative mapping? Relatedly, it remains unclear why the authors plan to code government actions in terms of gain/loss frame, information/emotion based content. There seems to be disconnect between these categories and the psychosocial variables assessed in WP 1 & 2, rendering a direct comparison difficult.
9. It may be helpful if the authors could provide (maybe as additional material or in a short paragraph) information about past and present public COVID-19 measures to enhance physical distancing in Ireland.
10. According to the abstract, one of three major goals of this research project is to optimize and tailor government communication. However, none of the WPs currently addresses how this goal is to be achieved in specific terms. The authors should provide details on how this is going to be implemented.

Is the study design appropriate for the research question?

Yes

Is the rationale for, and objectives of, the study clearly described?

Yes

Are sufficient details of the methods provided to allow replication by others?

Partly

Are the datasets clearly presented in a useable and accessible format?

Not applicable

Reviewer Expertise:

Psychology, self-regulation, motivation, goals, behavior change.

We confirm that we have read this submission and believe that we have an appropriate level of expertise to confirm that it is of an acceptable scientific standard, however we have significant reservations, as outlined above.

# Response

**Published:** 2020-12-04

**Authors:** Hannah Durand

**Affiliations:** NUI Galway, Ireland

*Durand et al. propose a quantitative and qualitative approach to understand the psychological and sociodemographic determinants of adherence to physical distance, as well as the degree to which government actions to increase physical distancing address these determinants adequately. As such, this research will likely contribute in a significant way to the scientific understanding and practical implementation of physical distancing measures during the response to COVID-19. While we endorse the overall approach of the authors, we identified some issues the authors should address to further strengthen their approach and its theoretical foundation.* 

 Response: We thank the authors for their constructive comments on our manuscript.  

*1.* *“This is an exploratory study; therefore, hypotheses are not specified a priori.”* 

*Why are no hypotheses specified? The authors outline empirical evidence regarding the variables used in their study and rely on two theoretical models to develop the research design, both of which could serve as a basis for formulating hypotheses. Moreover, the authors already seem to have hypotheses in mind when they refer to COVID-19-related beliefs/concerns and “at-risk groups”* *. * 

 Response: The aim of this study is to estimate the relative strength of a range of theoretically informed predictors on adherence to physical distancing guidelines in Ireland, not to test a set of predetermined hypotheses. There are a number of reasons why this decision was taken. Though considerable evidence regarding potential predictors of adherence to physical distancing is emerging, the quality of this evidence is as of yet unclear. Furthermore, physical distancing behaviour, which is best conceptualised as a suite of unique distinct behaviours, in the context of COVID-19 appears inherently different and arguably more complex than most health behaviours that existing theoretical models have been utilised to predict (e.g., adherence to a prescribed medical treatment, etc.). Therefore, we deemed it inappropriate to hypothesise a relationship between adherence to physical distancing behaviours and potential predictors at this time and in the context of an unprecedented health emergency. Instead this research is intended to provide an descriptive basis for future hypothesis generation and testing. This has been expressed in the manuscript.   

*2.* *The authors acknowledge the importance of automatic psychological processes as determinant for physical distancing adherence (e.g., habits). Yet, they solely refer to models addressing more reflective processes (e.g. beliefs) and remain silent about how these automatic processes are* *taken into account* *. This should be* *addressed, or* *explicated as a limitation of the proposed research. * 

 Response: The selection of models in this project, specifically the COM-B and Health Belief Model, was influenced by their use to guide the development of the iCARE study. As such there is limited scope to explore the role of automatic processes within WP1. WP2, however, will allow us the opportunity to qualitatively explore the role of automatic as well as reflective processes in adherence to physical distancing. This has been emphasised in the manuscript.  

*3.* *The authors describe very briefly two well-established models which contributed to their work in significant way. However, it might be useful if the authors outline the two models in a more detailed and a more distinctive manner (e.g. how the perceived severity of COVID-19 impacts physical distancing adherence). Especially the COM-B model should be treated in more detail because it is merely mentioned and not further explicated or discussed. As this research might be important for different fields of research (e.g. medicine, politics, sociology), a broader introduction of psychological theorizing seems appropriate. * 

 Response:  We have described the COM-B and Health Belief Model in greater detail in the manuscript.  

*4.* *Please operationalize the term “physical distancing” in a more distinct and detailed manner. Different measures can be implemented* *in order to* *keep physical distance among citizens. How is this variable measured? For instance, is it a single question or a questionnaire? And if it is a questionnaire, how will an overall physical distancing score be determined? * 

 Response:  Physical distancing will be operationalised in this research as a suite of behaviours encompassing maintaining two metres of physical distance between individuals, limiting one’s number of close contacts, and avoiding large gatherings. The extent of adherence to each of these behaviours will be measured using single items within the questionnaire. Given the distinct behaviours involved in physical distancing, an overall physical distancing score will not be calculated. Instead, each of the behaviours that make up physical distancing will be treated as unique outcome variables. This has been clarified in the manuscript.  

*5.* *The rationale for WP 2 remains somewhat fuzzy. The authors state that it is carried out to “explore in greater depth the barriers and facilitators of adherence”* *in an effort to* *complement the quantitative analyses in WP 1. However, it is unclear what the specific insights are that the authors hope to gain from the respondents in WP 2. What would be an outcome that goes beyond the quantitative measures? Alternatively, what do the quantitative measures miss that can only be addressed in the proposed interviews? This should be made more explicit, especially because WP 2 currently seems to make the smallest contribution to attaining the overall study goals. * 

 Response: There has been little if any qualitative investigation into adherence to physical distancing measures during COVID-19 to date. Given that both qualitative and quantitative research provide differential yet equally useful insights into human behaviour, utilising both methodologies in this project is a priority, as it will allow us to compare and contrast qualitative and quantitative findings and gain deeper insights overall. Furthermore, the qualitative interviews will allow us to explore other factors relevant to physical distancing generally (e.g., automatic factors) and in Ireland specifically (e.g., in relation to specific COVID-19 mitigation measures) that are beyond the scope of iCARE. This has been clarified in the manuscript.  

*6.* *The sample size considerations for WP 2 are currently not convincing. Answers from only 10-20 respondents could be biased by idiosyncratic experiences that might be hard to generalize to the public. On the other hand, it seems that the authors plan to recruit more participants if needed. They should specify the rules for extending/stopping data collection.* 

 Response: We appreciate that issues of sample size in qualitative research remain contentious. A general target of 10 – 20 participants has been suggested by Braun and Clarke, who developed the analytic approach we intend to use in WP2 (i.e., Thematic Analysis). However, we appreciate that this is simply a rule of thumb and not necessarily sufficient rationale for sample size targets in qualitative research. With a rough target of 10 – 20 participants in mind, evaluation of data adequacy, as per Vasileiou et al. (2018) and Guest et al. (2020), will be used to determine when to conduct no further interviews. This is the approach advised by Braun and Clarke (2019), over data saturation, for determining sample size in studies of this nature that utilise Thematic Analysis. Subsequent sampling to recruit more participants from under-represented sociodemographic groups will be done as needed to increase the representativeness of the sample, and to promote rigour and quality. However, it is important to note that generalisation of findings to the whole population, certainly in traditional statistical-probabilistic terms, is not the goal of this research. Statistically-informed approaches are not applicable to judge the value of qualitative research or claim that it lacks generalisability.  

*7.* *“This selection procedure, if necessary, will be informed by previous work of a similar nature and discussion among the research team to ensure that any selection biases in selecting public health for content analyses are limited.”* 

*Please outline the selection procedure in more detail or provide a reference where the procedure is described.* 

 Response: This admittedly vague passage was included before searching for physical distancing communications had begun in an attempt to provide a contingency plan for if the number of messages was unmanageable. Having started the process of selecting posters for inclusion in the analysis of WP3, it is now apparent that we have the resources to include all relevant unique communications. This has been amended in the manuscript.  

*8.* *In WP 3, the authors should be more specific about how they intend to map government actions and the psychosocial determinants of adherence studied in WP 1 & 2. For instance, will this be a quantitative or a qualitative mapping? Relatedly, it remains unclear why the authors plan to code government actions in terms of gain/loss frame, information/* *emotion based* *content. There seems to be disconnect between these categories and the psychosocial variables assessed in WP 1 & 2, rendering a direct comparison difficult.* 

 Response:  This has been clarified in the manuscript.  

*9* *.* *It may be helpful if the authors could provide (maybe as additional material or in a short paragraph) information about past and present public COVID-19 measures to enhance physical distancing in Ireland.* 

 Response:  Thank you for this suggestion. We feel that what may be most useful for the reader is to provide a link to the Government of Ireland Plan for Living with COVID-19, which delineates past and present measures to promote physical distancing in Ireland, but also will continue to be updated and provide real-time information after this article has been published. This has been added to the manuscript.  

*10.* *According to the abstract, one of three major goals of this research project* *is* *to optimize and tailor government communication. However, none of the WPs currently addresses how this goal is to be achieved in specific terms. The authors should provide details on how this is going to be implemented.* 

 Response: This is the intended outcome of WP3. This has been clarified in the manuscript.

# Peer review: Reviewer response for version 1

**DOI:** 10.21956/hrbopenres.14191.r27888

**Published:** 2020-09-15

**Authors:** Catherine Darker

**Affiliations:** 1 Department of Public Health & Primary Care, Trinity College Dublin, Dublin, Ireland

This is an interesting and valuable piece of research that has the potential to inform a key aspect of the public health response to COVID-19. The nesting of WP1 in an international study will bring many benefits. The inclusion of PPI involvement throughout is to be applauded.

1. Governments worldwide have implemented various forms of physical distancing measures with varying degrees of strictness and timings. The measures can include school/creche closures, cancellation of public events, stay at home orders, restrictions/limitations of distance from home orders, and international travel controls.
  How will the current study take cognizance of these types of physical distancing guidelines or is this research focused primarily on a 2m guideline between individual persons?
2. WP1 is utilising the methods and procedures outlined in a larger international project. The key advantage of this is Ireland contributing to an international project, while a key disadvantage is the restrictions that this might put on the study team in terms of deviations from the international protocol. Will the questions be piloted for an Irish population or does the team see that there is no need for this step? Will any questions be amended, excluded, or new additions be made?
3. The sample size for WP1 is unclear to me as there are two numbers presented: 1000 and 1200 participants. Please confirm which is the estimate and also what is the justification for same?
4. As a key driver for WP1 is to look at the determinants of adherence to physical distancing measures, please provide an understanding of how many of the 31 items focus on the respondent providing responses to their self reported adherence behaviours.
5. Please provide clarity on the recruitment process for WP2. It would seem to be a combination of purposive sampling (those that indicate greater or poor adherence to physical distancing) and quota sampling (e.g., "initial analyses will guide decision making regarding subsequent sampling"). Are those recruited for WP2 to complete a sub set of questions from WP1 to identify those that are compliant and those that are not? 
6. Will saturation be used as a stopping rule for WP2?
7. For clarity of reporting, I would propose that the researchers consider COREQ for WP2.
8. Can the researchers provide linkages to the literature with regards to the proposed procedural steps outlined for WP3? In, particular for the categorization and coding of the messages from the examples gathered.
9. From Figure 1 it is suggested visually that the WPs will inform each other in a sequential manner. However, as the researchers correctly point out they themselves will have to engage in reflexivity as they as citizens of the country will have been exposed to the public health messages since early Feb/March 2020. Would it be the case that the WP1 has both features of sequential and parallel designs? That these WPs will have an interactive effect? This is important in terms of interpretation and the multidimensional aspects of the project, patterns, and relationships.
10. Will each type of data (quantitative and qualitative) provided be considered equal in status?
11. Is the research including both the Republic of Ireland and Northern Ireland areas?

Is the study design appropriate for the research question?

Yes

Is the rationale for, and objectives of, the study clearly described?

Yes

Are sufficient details of the methods provided to allow replication by others?

Partly

Are the datasets clearly presented in a useable and accessible format?

Not applicable

Reviewer Expertise:

Health psychology, public health/population health, health behaviour change interventions, health promotion

I confirm that I have read this submission and believe that I have an appropriate level of expertise to confirm that it is of an acceptable scientific standard, however I have significant reservations, as outlined above.

# Response

**Published:** 2020-12-04

**Authors:** Hannah Durand

**Affiliations:** NUI Galway, Ireland

*This* *is an interesting and valuable piece of research that has the potential to inform a key aspect of the public health response to COVID-19. The nesting of WP1 in an international study will bring many benefits. The inclusion of PPI involvement throughout is to be applauded. * 

 Response: We would like to thank the reviewer for their positive and constructive feedback on our manuscript. We have responded to each of the reviewer’s comments in turn below. 

*1. Governments worldwide have implemented various forms of physical distancing measures with varying degrees of strictness and timings. The measures can include school/creche closures, cancellation of public events, stay at home orders, restrictions/limitations of distance from home orders, and international travel controls.* 

*How will the current study take cognizance of these types of physical distancing guidelines or is this research focused primarily on a 2m guideline between individual persons?* 

 Response: This research will focus primarily on measures such as maintaining two metres of physical distance between individuals, limiting one’s number of close contacts, avoiding large gatherings, etc. Other measures, for example closure of certain businesses, schools, etc., may emerge in the interviews conducted as part of WP2 as factors that affect an individual’s ability to physically distance, however these measures will not be our primary focus. The rationale for focusing on these behaviours specifically relates to greater extent of behavioural control (e.g., one must work from home if one’s workplace is closed by government mandate; therefore, adherence does not involve any decision-making on the part of the individual). This has been clarified in the manuscript.  

*2. WP1 is utilising the methods and procedures outlined in a larger international project. The key advantage of this is Ireland contributing to an international project, while a key disadvantage is the restrictions that this might put on the study team in terms of deviations from the international protocol. Will the questions be piloted for an Irish population or does the team see that there is no need for this step? Will any questions be amended, excluded, or new additions be made?* 

 Response: Our analysis in WP1 will focus on Wave 3 of the iCARE data in detail. Therefore Waves 1 and 2 may be considered a “pilot” of sorts in that each of these waves have allowed for potential issues with the survey to be addressed in subsequent waves. The study was designed to maximise the cross-contextual applicability of the measures included in the survey and to respond the new global issues as they emerged over the pandemic. This has also allowed for the inclusion of relevant questions as restrictions change over time. However, there has been no specific pilot of the iCARE questionnaire with the Irish population. The limitations of this approach have been clarified in the manuscript.   

*3. The sample size for WP1 is unclear to me as there are two numbers presented: 1000 and 1200 participants. Please confirm which is the estimate* *and also* *what is the justification for same?* 

 Response: The sample will be a nationally representative panel of 1000 participants. This has been amended for clarity in the manuscript.   

*4. As a key driver for WP1 is to look at the determinants of adherence to physical distancing measures, please provide an understanding of how many of the 31 items focus on the respondent providing responses to their* *self-reported* *adherence behaviours*. 

 Response: The survey asks participants to indicate which measures have been implemented by their government. Of these, 13 relate to physical distancing (e.g., Staying at least 2 metres away from others; Working from home; Self-isolating; Avoiding social gatherings). The remainder relate to hand hygiene, cough etiquette, wearing masks, etc. Participants are then asked to rate the extent to which they adhere to each of these individual measures (whether government mandated or not) on a four-point scale from ‘ *Most of the Time*’ to ‘ *Never*’. Participants are also asked to rate the extent to which certain government or local measures would convince them to practice physical distancing. The remainder of the survey addresses awareness, attitudes, and beliefs around COVID-19. The full survey tool is available via the Open Science Framework. This has been highlighted in the manuscript.  

*5. Please provide clarity on the recruitment process for WP2. It would seem to be a combination of purposive sampling (those that indicate greater or poor adherence to physical distancing) and quota sampling (e.g., "initial analyses will guide decision making regarding subsequent sampling"). Are those recruited for WP2 to complete a* *sub set* *of questions from WP1 to identify those that are compliant and those that are not? * 

 Response: Participants will be sampled purposively based on certain demographic characteristics (specifically, age, sex, and type of employment) that have been shown in the emerging literature to be predictive of adherence to physical distancing in the context of COVID-19. Participants will self-select into the study; however, if this results in an over- or under-representation of certain demographic groups then participants from those under-represented groups will be specifically targeted to ensure maximum variation in the sample. This has been clarified in the manuscript.  

*6.* ** *Will saturation be used as a stopping rule for WP2?* 

 Response: Evaluation of data adequacy, as per Vasileiou, Barnett, Thorpe, and Young (2018) and Guest, Namey, and Chen (2020), will be used to determine when to conduct no further interviews. This is the approach advised by Braun and Clarke (2019), over data saturation, for determining sample size in studies of this nature that utilise Thematic Analysis. This has been emphasised in the manuscript.    

*7.* ** *For clarity of reporting, I would propose that the researchers consider COREQ for WP2.* 

 Response: COREQ will be used in the reporting of WP2. This is stated under Dissemination and knowledge exchange plan: Academic dissemination.   

*8.* ** *Can the researchers provide linkages to the literature with regards to the proposed procedural steps outlined for WP3? In, particular for the categorization and coding of the messages from the examples gathered.* 

 Response: There is limited literature available to guide the procedural development of WP3. Our approach to WP3 is therefore relatively novel, having emerged primarily through conversation with the research team. The planned analysis will involve coding poster-based messages pertaining to physical distancing for Behaviour Change Techniques (BCTs) and mapping these onto the 26 Mechanisms of Action (Carey et al.) using the Theories and Techniques tool. We draw in part on previous work by Seppälä et al. (2018; https://doi.org/10.1186/s12889-017-4574-3), which involved a content analysis of policy papers in Finland, to guide the categorisation. This has been clarified in the manuscript.  

*9.* ** *From Figure 1 it is suggested visually that the WPs will inform each other in a sequential manner. However, as the researchers correctly point out they themselves will have to engage in reflexivity as they as citizens of the country will have been exposed to the public health messages since early Feb/March 2020. Would it be the case that the WP1 has both features of sequential and parallel designs? That these WPs will have an interactive effect? This is important in terms of interpretation and the multidimensional aspects of the project, patterns, and relationships.* 

 Response:  The reviewer raises an interesting point in this comment, however temporal elements of the project render the interactive potential of WPs 1 and 2 difficult. The nationally representative sample recruited to Wave 3 of iCare will have completed participation before recruitment of participants for the qualitative study begins. Therefore, we believe these work packages are best considered sequential as opposed to parallel.  

*10.* ** *Will each type of data (quantitative and qualitative) provided be considered equal in status?* 

 Response: We will treat both the qualitative and quantitative data as distinct but commensurate. Limitations on the conclusions we can draw from the iCARE survey (given it is an international survey as opposed to being developed specifically for an Irish context, and the cross-sectional nature of the nationally representative dataset) will be complemented by the rich qualitative data. The large scale nationally representative survey will allow us to gauge the attitudes and behaviours of a large nationally representative sample, while the qualitative study will allow us to drill down into key topics of interest in a much more in-depth way than a survey can allow. This has been emphasised in the manuscript.   

*11.* ** *Is the research including both the Republic of Ireland and Northern Ireland areas?* 

 Response: This research will include the Republic of Ireland only. This has been specified in the manuscript.
